# Ultrasound in Head and Neck Oncologic Perforator Flap Reconstruction: Recent Advancements in Preoperative Planning, Intraoperative Assessment, and Postoperative Monitoring

**DOI:** 10.7759/cureus.96557

**Published:** 2025-11-11

**Authors:** Shahriar Ghadirikhorzoghi, Parmida Massahi, Niyousha Mehrnoosh

**Affiliations:** 1 Plastic and Reconstructive Surgery, George Eliot Hospital NHS Trust, Nuneaton, GBR; 2 Biological Sciences, Aston University, Birmingham, GBR; 3 Plastic and Reconstructive Surgery, University Hospitals Birmingham NHS Foundation Trust, Birmingham, GBR

**Keywords:** doppler, duplex, flap monitoring, free flap reconstruction, head and neck neoplasms, microsurgery, oral cavity cancer, perforator flaps, ultrasound

## Abstract

Head and neck (H&N) oncologic reconstruction heavily relies on perforator flaps. Meticulous flap planning and early identification of vascular compromises are key to ensuring a high flap survival rate. Recent advancements in perioperative use of ultrasound have made it a versatile adjunct to traditional assessment methods to address their limitations. This comprehensive review aims to provide an updated narrative review of the evidence behind the current uses and frontier ultrasound modalities in the perioperative H&N oncologic flap reconstruction. The factors affecting the application of each ultrasound modality were analyzed. A comprehensive understanding of current and cutting-edge ultrasound techniques can aid in the development of institutional microsurgical assessment protocols and improve flap survival rates.

## Introduction and background

Perforator flaps have revolutionized head and neck (H&N) oncologic microplastic surgery, allowing for both form and function to be restored after tumor resection and chemotherapy/radiotherapy [1,2]. Free tissue transfer remains the gold standard for complex reconstructions in this region [3]. H&N presents one of the most challenging anatomical areas to microplastic surgeons due to altered vascular networks, limited access, cancer recurrence, and functional and aesthetic demands, which makes perioperative assessment of flap viability essential [2,4].

Ultrasound offers an inexpensive and safe imaging modality to overcome these challenges. In recent years, it has gained increasing prominence as a versatile adjunct in the management of perforator flaps, providing real-time insight into vascular integrity and perfusion [5].

Conventional handheld Doppler (HHD) and color Doppler ultrasound (CDU) have been used preoperatively to map perforator vessels and assess recipient site suitability, improving surgical planning and flap design precision [6,7]. More recent high- and ultra-high frequency ultrasound (HFUS/UHFUS) and contrast-enhanced ultrasound (CEUS) allow microscopic flap raise and 3D visualization of perforators, respectively [8,9]. Photoacoustic tomography (PAT) is a novel hybrid modality that produces accurate 3D vascular maps without the use of contrast agents [10]. Intraoperatively, modern color and spectral Doppler techniques enable real-time evaluation of anastomotic patency and hemodynamic parameters that directly correlate with flap viability [11]. Despite these advantages, traditional imaging modalities such as CT angiography (CTA) and magnetic resonance angiography (MRA) are preferred in some institutions, often overshadowing ultrasound due to operator dependency and high false positives [12].

Postoperatively, evidence for the use of ultrasound in flap monitoring is growing, yet it is underutilized [12]. Traditional clinical assessment remains the most common technique, but unlike ultrasound, it is often unable to detect early signs of vascular compromise, particularly in difficult-to-access or buried flaps [13-15]. With flap survival being critically dependent on early recognition of vascular compromise, ultrasound can be an invaluable tool as an adjunct to clinical assessment to avoid delayed management, irreversible flap loss, and further surgical intervention [16-18].

Despite its benefits, no comprehensive synthesis has yet explored the role of ultrasound across the pre-, intra-, and postoperative phases of H&N oncologic reconstruction. While CT and MRI have been studied considerably, the advantages of ultrasound in this area remain inadequately represented in the literature. This literature review aims to underline this gap by consolidating the recent progress in ultrasound modalities and their role in enhancing the outcomes of perforator flap reconstruction.

## Review

Methods

A comprehensive review of peer-reviewed literature published from 2000 to 2025 was conducted to evaluate the use of ultrasound in H&N oncologic reconstruction. The literature search utilized PubMed, EMBASE, and Google Scholar with different combinations of keywords such as “microsurgery”, “ultrasound”, “head and neck neoplasms”, “oral cavity cancer”, “perforator flaps”, “free flap reconstruction”, “flap monitoring”, “Doppler”, and “duplex”. The exact search syntax was: (("Head and Neck Neoplasms"[MeSH] OR "head and neck cancer" OR "oral cavity cancer" OR "facial cancer") AND ("Surgical Flaps"[MeSH] OR "free flap" OR "microvascular flap" OR "perforator flap" OR "flap reconstruction") AND ("Ultrasonography"[MeSH] OR ultrasound OR ultrasonography OR Doppler OR "color Doppler" OR duplex)) NOT (trauma OR congenital OR cleft OR animal OR cadaver). Forward and backward citation chaining was also used to obtain further related studies.

The review included the literature detailing the application of ultrasound across the preoperative, intraoperative, and postoperative phases of oncologic H&N perforator flap reconstruction, assessing conventional and novel ultrasound modalities. Articles that were irrelevant to microsurgery, non-peer-reviewed, outdated, and animal studies were excluded. A narrative approach was adopted to provide a broad overview and clinical interpretation of the evidence rather than an exhaustive systematic methodology, while highlighting key evidence gaps and outlining the priorities for future research.

Discussion

The first recorded H&N free flap reconstruction was performed by Panje et al. (1976) [19] for adenoid cystic carcinoma. Since then, the H&N free flap success rate has exceeded 85-90% [2]. Surgeons have shifted from conventional musculocutaneous and fasciocutaneous flaps to more refined, muscle-sparing perforator flaps, guided by high-resolution imaging methods such as CT angiography and ultrasound [1].

Currently, at every stage of the H&N perforator flap reconstruction, ultrasound plays a crucial role. With ultrasound, the donor and recipient tissues are accurately assessed preoperatively and carefully raised to include even the submillimeter perforator vessel [3,20,21]. It has proven beneficial for blood flow monitoring of the flap, allowing earlier detection of vascular compromise [18]. Combined with other tools such as 3D virtual surgical planning (VSP), indocyanine green (ICG), and AI, ultrasound offers both anatomical and functional information, showcasing its role as a reliable, practical, and versatile tool in reconstructive microsurgery [22-24].

In spite of these advantages, ultrasound must be compared to other established advanced modalities. For instance, although CDU has been shown to be more sensitive than CTA for perforator mapping of the anterolateral thigh (ALT) flap, it carries a higher false positive risk due to the detection of nearby great vessels [25]. Also, ultrasound limitations, including operator dependency, limited penetration in obese patients, time-intensive advanced techniques, and significant learning curves for surgical teams, must be acknowledged [12]. Various implications of ultrasound technology were reviewed across perioperative stages of flap reconstruction.

Preoperative applications

Advancements in preoperative techniques have reduced the time and cost for oncologic H&N reconstruction flap microsurgery [26,27]. The ALT flap, the radial forearm (RF) flap, and the osteocutaneous fibula (OF) flap have been the “Workhorse” for H&N reconstruction for several years due to ease of access and good pedicle caliber [5,28]. Regardless of the flap type, an ideal flap in this region should have high pliability, a reliable long pedicle, variable thickness, accurate definition of flap boundaries, and minimal donor-site morbidity to function in the 3D maxillofacial anatomy [2,4].

In addition, prior radiotherapy can lead to recipient endothelial damage and thrombosis, causing further complications and higher rates of flap failure [29,30]. Successful microvascular reconstruction in vessel-depleted necks demands surgical expertise, detailed anatomical knowledge, and the use of long, reliable perforators. Consequently, standardized treatment guidelines are still unavailable [31].

To overcome these challenges, preoperative uses of ultrasound predominantly involve assessment of donor perforator course, caliber, length, and existing atherosclerosis or anatomical variability, in addition to recipient vessel selection and 3D-VSP. Currently, HHD, CDU, HFUS, UHFUS, CEUS, PAT, and hybrid methods form part of the H&N surgeon’s armamentarium for optimizing oncologic flap viability [21,32,33].

HHD

HHD has been widely adopted for perforator mapping and recipient selection in H&N reconstruction due to its affordability and portable nature [6]. The probe is available in <10 MHz units with 15-20 mm deep peak sensitivity to facilitate reconstruction of vessels up to 0.5 mm in very thin intraoral paddles [34,35]. These advances led to the development of popular freestyle flaps, where flap delineation can be tailored around any perforator [36].

Pabst et al. (2023) [5] recently demonstrated in a European survey that Doppler sonography is still the most common form of preoperative imaging in most of the H&N free flap cases performed by participating oral and maxillofacial surgeons in Germany, Austria, and Switzerland. This popularity has likely been reinforced by the high clinical convenience of the device. In a prospective study, HHD was proposed by Tousidonis et al. (2021) [37] as a superior preoperative method of detecting vascular anomalies over the traditional Clinical Allen test for RF flap reconstruction. These findings were significant enough to change the preoperative flap of choice in 34 out of 87 oral cancer cases, underlining its role in preventing donor-site ischemia in the RF flap.

Despite the advantages, HHD is progressively losing ground to its competing modalities. In a comparative study by González Martínez et al. (2020) [6], its accuracy was lower than CTA and CDU, with a positive predictive value (PPV) of 88.6% compared to 100% for the latter methods. It is also limited by its unidirectional function, shallow effective depth range, inability to produce an accurate perforator map, and comparatively high false-positive rates [6]. It has been argued that it generates more false positives in thin patients due to detecting muscular perforators [38]. Additionally, HHD can cause suboptimal flap design and potential compromise of the pedicle by missing perforators traversing through fascial planes [39].

Nevertheless, HHD remains a valuable low-cost option to use as an adjunct to preoperative clinical assessment of the flap [5].

CDU

CDU has been influential in H&N oncologic reconstruction due to the incorporation of anatomy with color-coded vascular flow to generate a perforator and recipient map of vessels [40]. A systematic review by Cho et al. (2020) [12] showed that CDU has been heavily researched in the literature for perforator mapping use (39% of studies). Consequently, flap monitoring, pedicle dimension assessment, and recipient evaluation are significantly understudied.

The accuracy of CDU has been repeatedly demonstrated in the literature. In ALT flap planning, Debelmas et al. (2018) [7] showed approximately 90% accuracy in defining perforator location, type, and caliber using CDU. CDU also allowed for thinner and more pliable paddles suitable for intraoral reconstruction in a shorter operative time. CDU has been shown to map superficial circumflex iliac artery perforators as small as 0.5-0.7 mm reliably for buccal cancer reconstruction, supporting its use in complex cases [41]. A prospective study by Kehrer et al. (2020) [40] confirmed a 100% correlation between the intraoperative anatomy and CDU findings in a range of ALP perforator flap reconstructions, highlighting the high-grade precision of this tool in H&N perforator flap microsurgery.

In addition, Hong et al. (2022) [20] suggested CDU can be used effectively for recipient vessel selection, where multiple perforator vessels are identified and one is chosen that requires the least dissection. This study proposed a minimum peak systolic velocity (PSV) of 15-20 cm/s for flap survival. By calculating end-diastolic velocity (EDV), it can also provide the resistive index (RI) of the vessel ((PSV-EDV)/PSV). PSV and RI are indicators of flap perfusion and could determine the choice of perforator. With these values, CDU can therefore supply valuable information in decision-making for recipient vessel selection preoperatively. Similarly, in a retrospective study of 1616 patients undergoing deep inferior epigastric perforator (DIEP) flap, Ochoa et al. (2013) [42] showed that appropriate recipient veins can be screened to minimize the risk of venous congestion and ultimate flap failure. CDU can also mitigate risks of subclinical claudication of perforator flaps due to higher incidents of peripheral vascular disease in predisposed cancer cases, potentially preventing flap harvest abortion [43].

Comparative studies also suggest valuable benefits in using CDU compared to other modalities. In ALT perforator flap mapping, Haribhakti et al. (2021) [44] compared HHD with CDU and concluded that CDU can be used when HHD findings are inconclusive. Compared to CTA, CDU, with the added benefit of real-time hemodynamic information, provides higher levels of accuracy to locate perforators [21]. While some older studies prefer CTA over CDU [35,45], pooled analyses for ALT perforator identification advise that the highest sensitivity and PPVs for CDU are 95.7% and 94.3%, respectively. In complex cases and obese patients where ultrasound is unable to penetrate fully, CTA is preferable for extensive perforator course mapping [46].

Integrating CDU with other novel technologies has helped minimize flap mistakes even further. Recently, Illg et al. (2023) [47] proposed an innovative method of combining thermographic imaging with CDU to enhance perforator imaging capacity in ALT flap. The incorporation of both methods in perforator mapping showed that it accelerates CDU imaging by 90 to 130 seconds per exam by providing crucial information on angiosome location. In this method, the hotspots identified with thermography can provide supportive guidance for ultrasound techniques, hence improving operative turnover.

An alternative area of CDU use is the prediction of pedicle length. Surgeons commonly overestimate the flap volume to be 1.6 times larger than the intraoperative defect in anticipation of the flap atrophy following tumor resection [2]. Recent studies show that preoperative CDU can reliably predict ALT flap pedicle length for H&N reconstruction [48,49]. Łuczewski et al. (2019) [49] proposed a geometric formula for predicted pedicle length based on CDU measurements, which matched intraoperative results in 73.9% of cases, increasing to 84.1% with a margin of 5 mm. CDU also identified vascular variants precisely in 97% of patients. These findings underscore CDU as a versatile and accurate gadget to enhance preoperative planning by confirming pedicle suitability and rectifying anatomical differences.

Barriers exist in the widespread incorporation of this technology, including limited accessibility, operator dependency, and a steep learning curve. Interestingly, evidence by Bajus et al. (2023) [50] supports the learning curve plateau after performing a limited number of cases with CDU to reach a CTA-level perforator location accuracy. Nevertheless, CDU is suggested as one of the most multipurpose imaging modalities for preoperative planning of oncologic H&N microsurgery, combining anatomical, functional, and safety benefits unmatched by other modalities [49].

HFUS/UHFUS

With increasing demands in high-resolution imaging of superficial structures in H&N oncologic reconstruction, the application of HFUS (>15 MHz probe) has been amplified. In this region, the objective often shifts from replacing volume to precise resurfacing. HFUS aids the surgeons’ preoperative decision-making through obtaining real-time data on flap physiology up to 30 mm depth, anticipating precise anatomical variations of 50-150 µm, and expediting dissection [8,51]. In a recent retrospective study, Lee and Lee et al. (2024) [52] demonstrated that using HFUS can significantly decrease operative time in superthin ALT or pure-skin SCIP flaps. This was due to faster flap elevation compared to HHD. In addition, it was suggested that preoperative use of HFUS may reduce partial flap necrosis rates by allowing the choice of the least invasive perforator, resulting in safer harvest. Nevertheless, this field is relatively novel, and further comprehensive studies are warranted.

In addition, UHFUS (30-70 MHz probe) provides a reliable axial resolution of up to 30 µm. This innovation has facilitated the visualization of very small superficial capillary perforators and intradermal plexus for high-quality submillimeter flap raise [8]. Visconti et al. (2020) [53] showed that UHFUS provides superior visualization of microvascular anatomy for thin, superthin, and pure skin perforator flap ALT or SCIP flap planning. This allowed the proposition of a practical classification method of the perforator anatomy when the perforator penetrates the Scarpa and the superficial fascia [27,54]. The preoperative results also had 100% correlation with intraoperative findings, which signal increased surgical efficiency and mitigate flap-related morbidity in oncologic reconstruction.

UHFUS presents unique advantages over traditional CDU. CDU is often limited by its superficial low resolution and higher false-positive pedicle recognition. In contrast, UHFUS allows differentiation of arteries and veins, as arterial pulsations and venous mosaic flow patterns are portrayed at high resolution. In essence, flap design can consistently trace distal vessels of the vascular pedicle into the dermis, which may be a factor in the absence of partial flap necrosis using this technology [55]. However, this method is accompanied by an even steeper learning curve, limited depth, and considerable financial burdens, contributing to its limited availability [8].

CEUS

Su et al. (2013) [32] first introduced CEUS, which utilizes an intravenous microbubble contrast agent, improving vascular visualization and image quality for perforator mapping [9]. CEUS has been used across various H&N perforator flaps where vascular structure is variable and complex, such as the thoracic branch of the supraclavicular artery flap. In such cases, CEUS coupled with 3D reconstruction showed higher specificity (100%) and sensitivity in perforator identification over CDU [56]. Although both CEUS and CDU can detect small perforators as small as 0.5 mm, CEUS offers exceptional flow contrast, sensitivity, and delineation of subtle vascular pathways. Moreover, it provides hemodynamic information without ionizing radiation or nephrotoxic contrast, unlike CTA or MRA, making it more suitable for planning complex microsurgeries [56]. Despite the advantages, this technique is time-consuming, requires a skilled operator to perform, and has less consistent accuracy in vessel caliber evaluation over CDU, which can hinder its widespread use [56].

B-flow BCEUS is an advanced method of CEUS application that uses B-flow imaging to provide detailed and dynamic flow directions. B-flow is a non-Doppler ultrasound modality that aids visualization of blood flow by enhancing blood cell signals. This was employed by Zinser et al. (2022) [57] in DIEP flap planning, and it proved to be a feasible alternative to CTA in terms of precision and mapping of the fascial penetrating perforator compared to intraoperative findings (mean CEUS deviation ≈ 4.6 mm, mean CTA deviation ≈ 2.9 mm). However, they also mention that all of the BCEUS examinations were done by a highly specialized sonographer. Indicating potential bias behind comparable accuracies to CTA. This also came at the cost of a much longer examination compared to CTA. Nevertheless, other studies also suggest the advantage of BCEUS to detect skin exit points with 96% accuracy and a 3 mm mean precision in tracing the subcutaneous and epifascial courses of perforators, surpassing CEUS, CDU, and B-flow alone [58]. This evidence proves BCEUS can be a valuable adjunct in complex microsurgeries for optimizing flap design, reducing intraoperative uncertainty, and enhancing surgical confidence.

Overall, although CEUS and BCEUS have significantly enhanced vascular mapping and intraoperative safety, they may not provide practical and reliable results alone and should be conjugated with other techniques such as CTA and CDU to offer a comprehensive, multimodality approach for the preoperative phase of microsurgical H&N reconstruction [57].

Use of Ultrasound Technology in 3D-VSP

3D-VSP has been popularized in H&N flap reconstruction with widespread use of CTA and MRA. Computer-aided design and computer-aided manufacturing technology integration with CT scans is being established in H&N for perforator mapping and 3D printing of prostheses, particularly for OF flaps in mandibular defects [59,60]. While CTA provides 3D bone and vascular visuals, it is expensive, uses high levels of ionizing radiation, and lacks hemodynamic data. Its strength in bone imaging contrasts with its low tumor margin definition, which may require MRI coupling for tumor margin assessment. This can incorporate further registration errors [2,59,61]. Given the limitations of CT-based VSP, other imaging modalities have been proposed.

3D ultrasound technology has been demonstrated to be a viable option for 3D-VSP. In this method, the scanning is performed with or without contrast and can be exported as 360-degree live video content. In an extensive prospective cohort study, Shen et al. (2019) [23] used 3D-CDU for perforator navigation of H&N reconstruction and confirmed its reliability, with all of the mapped perforators verified intraoperatively, and false-negative rates were only 1.6%. Interestingly, they did not observe a difference with or without a contrast in mapping accuracy, although the contrast still has its role in improving the echo of ultrasound and helps with locating smaller vessels. Nevertheless, similar accuracies have been observed with older studies using contrast [32]. This proves the uniformity of this method in the preoperative stage and can be a reliable option for small flap reconstruction in the H&N region.

Hybrid methods have also been used to incorporate ultrasound into H&N VSP workflows. Berrone et al. (2016) [62] manually registered the geometrical location of perforators obtained by an HHD into a 3D-VSP interface for OF cases. No flap failure occurred, and in only four out of six cases, there was a good correlation between perforator location and the taken bone segment. This may highlight HHD’s limited accuracy compared to CTA; however, larger prospective literature is needed to validate these findings.

In addition to flap design, a recent study by Kaltoft et al. (2024) [63] showed advancements in oral tumor staging and margin assessment by 3D intraoral ultrasound. This technology surpassed MRI in invasion depth precision, with histopathologic analysis of resected specimens corresponding strongly with intraoperative findings (mean US deviation ≈ 1.0 mm, mean MRI deviation ≈ 1.9 mm). This real-time imaging capability can usher in intraoperative decisions, improving oncologic safety and outcomes.

PAT

PAT is an emerging hybrid technique using laser-ultrasound technology for vascular imaging in reconstructive microsurgery. Its emitted near-infrared laser pulses are absorbed by hemoglobin to generate ultrasound waves through thermoelastic extension. This produces 3D vascular maps without using contrast agents [10,64]. In a prospective study, Tsuge et al. (2020) [10] showed PAT can have similar precision to CDU for assessing supra-fascial vessels and even be superior for envisioning oblique and horizontal perforators for the H&N region. These early findings indicate a potential role for PAT in conjunction with CDU in preoperative assessment of the flap.

Hardware and software integrations have further expanded the clinical role of PAT. Tsuge et al. (2020) [10] developed a sterilizable transparent cover for aligning preoperative PAT maps with corresponding anatomy, but minor alignment errors were reported. Suzuki et al. (2021) [65] used ICG lymphography with PAT to enhance this technique. Visuals were shown to the surgeon via a head-mounted display, which accomplished strong intraoperative correlation but required lengthy calibrations and was often not feasible in routine practice.

Xiao et al. (2025) [66] studied the integration of neural networks to improve the clinical applications of PAT. The H&N region has nonuniform shapes that sensors may not entirely surround the flap, causing limited view and image blurring. This model could accurately approximate implicit spatial representations. These innovations highlight the application of multimodal systems combining PAT, ultrasound, and augmented reality to enhance preoperative accuracy.

PAT provides high-resolution 3D images of superficial vessels without radiation, but it is a complementary technique rather than a replacement. CDU and HFUS are still preferred for depth of penetration, real-time hemodynamics, and arteriovenous distinction [10,67]. Collectively, PAT and CDU can provide a more holistic and safer approach to preoperative planning in H&N flap reconstruction.

A qualitative summary of preoperative assessment modalities is presented in Table 1.

**Table 1:** Comparative characteristics of preoperative assessment modalities BCEUS, B-flow contrast-enhanced ultrasound; CEUS, contrast-enhanced ultrasound; CDU, color duplex ultrasound; CTA, CT angiography; EDV, end-diastolic velocity; HFUS, high-frequency ultrasound; HHD, handheld Doppler; MRA, magnetic resonance angiography; PAT, photoacoustic tomography; PSV, peak systolic velocity; RI, resistive index; UHFUS, ultra-high-frequency ultrasound

Modality	Reference(s)	Imaging capacity	Reported performance	Limitations	Cost
HHD	González Martínez et al. (2020) [6]	<10 MHz acoustic probe; 15-20 mm depth peak sensitivity; acoustic detection of perforator location	Basic perforator localization; suitable for quick screening	No anatomical detail; high false positives; operator-dependent	Very low cost
CDU	Hong et al. (2022) [20]; Kehrer et al. (2020) [40]	Multifrequency linear transducers of 6-15 MHz; variable depth; reliably identify vessels ≥0.5 mm diameter; measure PSV, EDV, RI, and branch patency	Tissue differentiation based on echogenicity; highly concordant with intraoperative anatomy; high sensitivity for perforator mapping	Requires optimized settings and a trained operator; superficial low resolution	Low to moderate cost
HFUS	Crisan et al. (2022) [51]; Lee and Lee et al. (2024) [52]	15-30 MHz probe; 2-3 cm depth; 50-150 µm resolution; superficial perforator anatomy for thin flaps	Faster flap elevation time and necrosis rates	Learning curve; limited penetration depth	Low cost
UHFUS	Hayashi et al. (2022) [8]; Visconti et al. (2020) [53]	30-70 MHz probe; 3-10 mm depth; up to 30 µm resolution; microvascular/lymphatic anatomy down to the dermal level	High agreement with intraoperative anatomy	Learning curve; extremely shallow penetration depth; operator-dependent	Moderate cost
CEUS/BCEUS	Gao et al. (2016) [56]; Zinser et al. (2022) [57]	Variable probe; dynamic visualization of flow, epifascial course, and skin-point mapping; reliably identify vessels ≥0.5 mm diameter	High accuracy; improved submillimeter precision	Time-consuming; technically demanding; experienced operator required	Moderate cost; contrast agent required
PAT	Tsuge et al. (2020) [10]; Shimizu et al. (2022) [64]	6-21 mm maximal depth recognition; 3D vascular mapping	High agreement with intraoperative anatomy within 1 cm; better for envisioning oblique and horizontal perforators	Complementary to other modalities, time-consuming, difficult to differentiate artery from vein, limited view	Moderate cost
CTA	Lo and Lin (2023) [59]; Myers et al. (2021) [60]	Complete perforator course from origin to skin surface; ≤1 mm slices	Gold-standard accuracy; excellent 3D reconstruction	Radiation and contrast exposure; requires an imaging suite	High per-study cost
MRA	Saito et al. (2012) [61]	Comprehensive vessel mapping without radiation; ~0.5-1.0 mm slices	Highly accurate; superior soft-tissue contrast	Long acquisition time; high cost; limited availability	High cost

Intraoperative applications

Intraoperative factors, such as ischemic duration, microvascular technique, and flap monitoring, are essential for flap viability. Reducing reperfusion injury and ensuring graft survival requires prompt revascularization and hemostasis. Intraoperative monitoring techniques, such as clinical judgement, ICG angiography, and ultrasound Doppler, are valuable tools for measuring perforator flow and guiding surgical adjustments [5].

Real-Time Assessment of Anastomotic Patency and Flap Planning

Modern CDU technology allows intraoperative assessment of hemodynamic parameters that have substantial predictive value in flap surgery. Several studies have found that PSV directly correlates with successful perforator dissection, and a velocity of 20 cm/s is a reliable threshold for flap harvest [11,20]. These calculations are utilized by the surgeon intraoperatively to change the perforator if a more viable vessel is found.

HHD is a simple and affordable tool to assess flow, but it is limited in identifying low-flow venous signals or diffuse capillary perfusion. Accuracy can also vary with operator skill and body habitus [68]. Recently, enhancing HHD ultrasound with modalities such as ICG angiography to enable simultaneous visualization of macrovascular patency (ultrasound) and microcirculatory perfusion (ICG) has been proposed. Kim et al. (2024) [22] demonstrated that this combined approach in ALT flap reconstruction for extensive scalp defects may improve intraoperative judgment and general reconstructive precision. Although the combination reduced complication rates, this was not statistically significant (75% vs. 25%, P = 0.607). In addition, surgeons need to allow more time to perform both techniques. Larger prospective studies are required to assess the potential benefits of this method.

Dynamic Flap Planning

Advanced intraoperative applications, particularly dynamic flap planning, have been described in the literature [12,69]. In this technique, preoperative markings are reconfirmed intraoperatively, and new perforators may be chosen if they have greater potential. For instance, Song et al. (2019) [69] showed that the direction of propeller flap rotation significantly changes flow velocity and volume, with intraoperative CDU to aid surgeons in selecting the rotation arc that is the highest perfusion-preserving. In practice, flow velocity is measured with duplex ultrasound after a 180-degree flap rotation to choose the side with greater perfusion [12]. These findings underline ultrasound’s ability to confirm vascular patency and provide real-time intraoperative decision-making guidance.

Cancer Margin Evaluation

Cancer resection fields may contain malignant cells and increase the risk of local recurrence and flap failure at the junction between the transferred flap and resident tissue. Hence, the reconstructive flap must be free of malignancy [2]. Recent advances in 3D ultrasound have provided valuable tools for improving the safety of H&N oncologic flap reconstruction [70,71]. For instance, a prospective study of surgeons performing transoral 3D ultrasound of oropharyngeal squamous cell carcinoma showed a strong correlation between histopathology of tumor and margin measurements (r = 0.76-0.85). This enabled the correct prediction of tumor location with 93% sensitivity compared to 76% with panendoscopy or 28% with MRI [71,72]. The scanning took only eight minutes on average and demonstrated a significant reduction in the risk of flap failure from cancer recurrence.

Regardless of challenges with workspace, operator experience, and sterility, advances in smaller robot probes and AI-enhanced software are expected to integrate 3D ultrasound into microsurgery for improved reconstructive precision and oncologic surveillance [72].

A qualitative summary of intraoperative assessment modalities is presented in Table 2.

**Table 2:** Comparative characteristics of intraoperative assessment modalities CDU, color duplex ultrasound; EDV, end-diastolic velocity; HHD, handheld Doppler; ICG, indocyanine green; PSV, peak systolic velocity; RI, resistive index

Modality	Reference(s)	Imaging capacity	Reported performance	Limitations	Cost
Clinical judgment	Yoshida et al. (2021) [11]; Hong et al. (2022) [20]	Visual assessment of flap color, turgor, bleeding, and capillary refill	Subjective but essential for immediate intraoperative feedback	Operator-dependent; very limited sensitivity for buried flaps	No cost
HHD	Phillips et al. (2016) [68]	<10 MHz acoustic probe; 15-20 mm depth peak sensitivity; acoustic signal of macrovascular flow	Helpful in detecting arterial patency; limited for low-flow or venous signals	Operator-dependent; no visual detail; no assessment of microcirculation	Very low cost
HHD + ICG angiography	Kim et al. (2024) [22]	Simultaneous visualization of macrovascular and microvascular perfusion	Improved intraoperative precision and perfusion assessment; may reduce complication rates	Time-consuming; requires ICG dye and additional equipment	Moderate cost (ICG agent and imaging system)
CDU	Cho et al. (2020) [12]; Song et al. (2019) [69]	Real-time hemodynamic assessment (PSV, EDV, and RI) of perforators and recipient vessels	PSV ≥ 20 cm/s correlates with flap success; aids vessel selection and perfusion optimization	Requires experience and proper calibration; limited access in deep or narrow fields	Low to moderate cost
3D ultrasound	Bekedam et al. (2021) [70]; Garset-Zamani et al. (2025) [71]	High-resolution imaging of tumor margins and flap-tissue interface	93% sensitivity for tumor location vs. 28% with MRI; mean scan time: eight minutes	Requires space, probe maneuverability, and an experienced operator; not yet standardized	Moderate to high cost

Postoperative applications

Although overall success rates for free flaps in the H&N region exceed 95%, the need for revision surgery remains significant at 10-12%. Extensive retrospective studies suggest H&N oncologic flap reconstruction is associated with higher rates of flap failure [73,74], with early flap loss due to vascular compromise and late flap loss mainly caused by postoperative radiotherapy [2]. In spite of well-documented complications, there is limited literature detailing the use of technological advancements in the postoperative phase of flap reconstruction [12].

Clinical examinations such as flap color, edema, warmth, capillary refill, and pinprick hemorrhage are still most commonly used for postoperative monitoring and evaluating flap perfusion [14]. Clinical findings rely upon observers, which can lead to late ischemia detection and are not suited for buried flaps [15,75]. With over 95% of flap compromises occurring within the first 72 hours postoperatively [76], adjunct methodology would be required to prevent further surgical interventions. Consequently, ultrasound techniques have been proposed to improve accuracy and reliability, such as HHD, CDU, implantable Doppler, and, more recently, AI integration [17,18,24,77].

HHD

HHD is one of the techniques for postoperative H&N free flap monitoring, as it is simple, portable, and can reach difficult anatomical areas via a small probe [78]. This technique not only decreases the flap failure risk through real-time assessment of blood flow but can also provide valuable information about other complications such as hematomas, seromas, and soft tissue infections [14]. An extensive retrospective study by Hosein et al. (2016) [14] indicates higher sensitivity of HHD compared to implantable Doppler probes when used by a skilled operator.

However, this technique has a few significant disadvantages in monitoring flaps, including intermittent assessment of vascular blood flow instead of continuous monitoring. In addition, postoperative compact and altered anatomy makes distinguishing arterial from venous flow and pedicle from adjacent recipient vessels difficult. These false positive results will be made more complex by anastomotic couplers and buried flap positioning [14,15]. Regardless, this method is widely available in conjunction with more precise monitoring methods.

CDU

External CDU can utilize color flow and spectral Doppler images from both donor and recipient to assess the anastomotic patency and flap viability by placing the probe on top of the reconstructed tissue [15,79]. This method can also provide objective perfusion assessment in buried flaps due to higher penetration compared to conventional monitoring methods, such as pinprick. The probes can be used to estimate flow resistance and produce a pulsatility index, which is associated with venous compromises; hence, it is a widely used adjunct to other flow-measuring techniques [15].

Availability of this device at the patient’s bedside may augment routine nurse-led postoperative flap monitoring for the first postoperative week [80]. In addition, in a prospective study, Shinomiya et al. (2020) [81] suggested reducing the false positives of clinical assessment to zero, where no unnecessary flap exploration is performed upon regular CDU monitoring. Although promising, further multicenter research is needed for validation of these findings.

On one hand, similar to HHD, CDU is noncontinuous and has been shown to struggle with accurately distinguishing perforator signals from background major vessels such as jugular veins and carotid arteries postoperatively. These are vital drawbacks in monitoring flap survival in the vulnerable H&N tissues [79,81].

On the other hand, the availability of modern portable ultrasound even in resource-limited settings has been shown to improve postoperative monitoring. A study by Wood et al. (2021) [82] showed that during an H&N free flap reconstruction camp, solely the presence of CDU was sufficient for successful monitoring without the need for other techniques. Applying the probe right at the anastomotic site immediately shows arterial triphasic waveforms and venous continuity, while the absence of the signals or irregularity would warrant immediate action. Additionally, this method can be more cost-effective in the long term compared to other equipment for flap monitoring due to reusability, lower invasiveness, and training costs [80]. However, a detailed financial comparison is still needed to explore this matter.

These findings can highlight the potential reliability and adaptability of CDU for postoperative vascular assessment [82]. It would seem appropriate for future research to focus on clear outcome measures in the evaluation of the CDU technique in clinical practice.

Implantable Cook-Swarts Doppler (CSD) Probe

The implantable CSD probe is an invasive method that utilizes a 20 MHz ultrasonic silicone cuff that encircles the vascular pedicle to produce continuous hemodynamic data. This can be an advantage in H&N reconstructive surgery, particularly for monitoring buried or poorly accessible free flaps, where traditional clinical examination is challenging [16]. The device was first introduced by Swartz and aims to detect vascular compromise before clinical signs appear by providing real-time information on blood flow within anastomosed vessels [83].

A prospective study by Hayler et al. (2021) [84] demonstrated its effectiveness in early detection of vascular compromise, with the CSD identifying compromised flaps up to 60 minutes earlier than microdialysis. These results were in accordance with older studies on implantable Doppler technology [15,85]; for instance, a systematic review by Chae et al. (2015) [15] included six comparative studies to demonstrate that the implantable Doppler probe likely improved flap salvage rate compared to clinical monitoring. However, there is a lack of recent high-level evidence literature to support its current role in H&N microsurgery.

Drawbacks include the introduction of a foreign body near the pedicle, high false positives, and the inability to assess end-capillary perfusion [80,86,87]. Like the HHD, it measures only pedicle flow and may fail to detect microcirculatory compromise or nonarterial causes of flap failure [87].

CEUS

This method provides selective imaging of blood volume in the microcirculation up to 4 cm deep, as the controlled microbubble dissolution and the following restoration allow for a dynamic assessment of blood velocity. Its ability to provide direct angle-independent visual information on tissue perfusion at a microvascular level makes it particularly suitable for the H&N region. It can monitor both buried and nonburied flaps, which is essential given the high prevalence of buried flap use in H&N reconstruction [88,89]. Furthermore, using CEUS as confirmatory imaging may reduce unnecessary reexplorations [78].

However, it is still operator-dependent, lacks the ability to provide continuous monitoring, and requires integration of external software, all of which make it susceptible to delayed decision-making in time-sensitive ischemic events [90]. Although early studies are promising, general caution is advised, as the evidence in this area is limited and requires further comprehensive research.

A qualitative summary of postoperative assessment modalities is presented in Table 3.

**Table 3:** Comparative characteristics of postoperative assessment modalities CEUS, contrast-enhanced ultrasound; CDU, color Doppler ultrasound; HHD, handheld Doppler

Modality	Reference(s)	Imaging capacity	Reported performance	Limitations	Cost
Clinical examination	Chae et al. (2015) [15]	Visual and tactile evaluation of flap color, warmth, turgor, and capillary refill	Widely practiced; useful for superficial flaps	Observer-dependent; unsuitable for buried flaps; can delay ischemia detection.	No cost
HHD	Hosein et al. (2016) [14]	<10 MHz acoustic probe; 15-20 mm depth peak sensitivity; audible signal of arterial and venous flow through a small probe	High sensitivity when used by experienced operators; detects flow in difficult-to-reach areas	Intermittent monitoring only; high false positives; influenced by anatomy	Very low cost
CDU	Cuthbert et al. (2018) [80]; Shinomiya et al. (2020) [81]; Wood et al. (2021) [82]	Color and spectral Doppler imaging to assess anastomosis and perfusion	Reliable assessment of buried flaps; provides flow resistance and pulsatility index; feasible for bedside use	Limited microcirculation assessment; high false positives	Low to moderate cost
Implantable Doppler probe (Cook-Swartz)	Chae et al. (2015) [15]; Hayler et al. (2021) [84]	Continuous real-time measurement of pedicle blood flow	Detects vascular compromise up to 60 minutes earlier than microdialysis; improves flap salvage rates	Invasive; foreign body near pedicle; high false positive; cannot assess end-capillary perfusion	High cost (device + implantation)
CEUS	Geis et al. (2016) [89]; Mueller et al. (2017) [90]	Dynamic visualization of microvascular perfusion; penetration depth 10 cm	High sensitivity for buried flaps; provides quantitative perfusion data	Operator-dependent; noncontinuous; invasive, less mobile; limited contrast availability	Moderate cost; contrast agent required

Use of AI in Perioperative Stages of Reconstruction

AI is a promising complement to the ultrasound technique in perforator mapping and flap monitoring. Analyzing large datasets and identifying subtle imaging trends allows one to conclude predicted outcomes. Machine learning (ML) algorithms can already predict the outcome of H&N flap reconstruction accurately based on patient demographics, aiming to improve identification of high-risk patients [91].

Novel ML models are being used for analyzing imaging modalities in the reconstructive field. For instance, recently Mavioso et al. (2020) [92] used computer software to automatically map the perforator vessels of the DIEP flap from CTA images. Results showed a significant increase in key vessel detection by software compared to manual, with 97.7% accuracy, supporting that ML techniques can enhance the microsurgical preoperative planning. Although this study solely used CTA images for software training, new ML models such as Faster-RCNN with the Inception-ResNet-v2 deep-learning framework have been integrated with breast ultrasound technology, which can be transferred to other anatomical modalities such as H&N [93].

Accurate recognition of vascular abnormalities, tumors, and early complications such as infection, hematoma, or implant malposition through different learning modalities has been highlighted in the literature [93]. Consequently, AI may also be capable of mitigating the challenges of ultrasound application, including variability in techniques, operator dependency, and the lack of standardized protocols of flap volume or perfusion, resulting in more consistent and cost-effective monitoring practices [24]. Literature in this area still lacks proficiency, warranting further validation through larger, prospective, multicenter studies that consider factors such as the surgeon’s experience.

## Conclusions

Ultrasound is a growing technology in H&N oncologic perforator flap reconstruction in all of the surgical steps, from preoperative flap planning to postoperative monitoring. This method provides accurate information on perforator mapping, vessel quality assessment, and early perfusion compromise detection. It can provide real-time and noninvasive vascular visualization to improve flap salvage rate and functionality. Various ultrasound modalities are available, including HHD, CDU, HFUS, UHFUS, CEUS, and PAT, with their unique abilities, making ultrasound an adaptable method for overcoming H&N complex reconstruction challenges.

Despite all the advancements, the literature is limited primarily to small cohort studies and is fragmented. Robust prospective research is required to clarify the ultrasound’s value compared to other existing imaging and monitoring techniques. Looking ahead, integrating other modalities such as ICG, CTA, and AI with ultrasound has the potential to enhance ultrasound’s drawbacks in all phases of H&N reconstruction, such as operator dependency and high false positives. This could be achieved via automated vascular detection, 3D-VSP, flap perfusion prediction models, and image refining techniques, making it a crucial hotspot of innovation in reconstructive microsurgery.
